# Sex Dimorphic Associations of Gestational Diabetes Mellitus With Cord Plasma Fatty Acid Binding Protein 4 and Estradiol

**DOI:** 10.3389/fendo.2021.740902

**Published:** 2021-09-21

**Authors:** Xin Liu, Tao Zheng, Ya-Jie Xu, Meng-Nan Yang, Wen-Juan Wang, Rong Huang, Guang-Hui Zhang, Yu-Na Guo, Jun Zhang, Fengxiu Ouyang, Fei Li, Zhong-Cheng Luo

**Affiliations:** ^1^Ministry of Education-Shanghai Key Laboratory of Children’s Environmental Health, Early Life Health Institute, Department of Developmental and Behavioral Pediatric & Child Primary Care, Xinhua Hospital, Shanghai Jiao Tong University School of Medicine, Shanghai, China; ^2^Lunenfeld-Tanenbaum Research Institute, Prosserman Centre for Population Health Research, Department of Obstetrics and Gynecology, Mount Sinai Hospital, Institute of Health Policy, Management and Evaluation, Dalla Lana School of Public Health, Faculty of Medicine, University of Toronto, Toronto, ON, Canada; ^3^Department of Obstetrics and Gynecology, Xinhua Hospital, Shanghai Jiao-Tong University School of Medicine, Shanghai, China; ^4^Department of Clinical Assay Laboratory, Xinhua Hospital, Shanghai Jiao-Tong University School of Medicine, Shanghai, China; ^5^Department of Obstetrics and Gynecology, International Peace Maternity and Child Health Hospital, Shanghai Jiao-Tong University School of Medicine, Shanghai, China; ^6^Brain and Behavioral Research Unit, Shanghai Institute of Pediatric Research, Xinhua Hospital, Shanghai Jiao Tong University School of Medicine, Shanghai, China

**Keywords:** gestational diabetes mellitus (GDM), fatty acid binding protein 4 (FABP4), adiponectin, testosterone, estradiol (E2), sex dimorphism

## Abstract

Fatty acid binding protein 4 (FABP4) has been associated with insulin resistance. Gestational diabetes mellitus (GDM) impairs fetal insulin sensitivity. Female newborns are more insulin resistant than male newborns. We sought to evaluate the association between GDM and cord blood FABP4, and explore potential sex dimorphic associations and the roles of sex hormones. This was a nested case-control study in the Shanghai Birth Cohort, including 153 pairs of newborns in GDM *vs*. euglycemic pregnancies matched by infant sex and gestational age at delivery. Cord plasma FABP4, leptin, total and high-molecular-weight adiponectin, testosterone and estradiol concentrations were measured. Adjusting for maternal and neonatal characteristics, cord plasma FABP4 (Mean ± SD: 27.0 ± 19.6 *vs*. 18.8 ± 9.6 ng/mL, P=0.045) and estradiol (52.0 ± 28.6 *vs*. 44.2 ± 26.6, ng/mL, P=0.005) concentrations were higher comparing GDM *vs*. euglycemic pregnancies in males, but similar in females (all P>0.5). Mediation analyses showed that the positive association between GDM and cord plasma FABP4 in males could be partly mediated by estradiol (P=0.03), but not by testosterone (P=0.72). Cord plasma FABP4 was positively correlated with total adiponectin in females (r=0.17, P=0.053), but the correlation was in the opposite direction in males (r=-0.11, P=0.16) (test for difference in r, P=0.02). Cord plasma FABP4 was not correlated with leptin in both sexes. The study is the first to demonstrate sex-dimorphic associations between GDM and cord plasma FABP4 or estradiol, and between FABP4 and adiponectin in newborns. GDM may affect fetal circulating FABP4 and estradiol levels in males only.

## Introduction

Gestational diabetes mellitus (GDM) is characterized by glucose intolerance with first recognition in the 2^nd^ half of pregnancy, affecting 3%-25% of pregnancies worldwide (1, 2). The offspring of mothers with gestational diabetes are at elevated risks of insulin resistance and type 2 diabetes (3, 4). The mechanisms linking GDM in early life “programming” the vulnerability to type 2 diabetes remain unclear.

A number of adipokines are involved in the regulation of insulin sensitivity, most notably leptin and adiponectin (5). GDM has been associated with impaired insulin sensitivity, elevated leptin and decreased adiponectin concentrations in the newborns (6–9). However, little is known about whether GDM may affect circulating levels of other adipokines in early life.

Fatty acid binding protein 4 (FABP4) is an adipokine involved in the transport of fatty acids to specific organelles in the cell (8). It has been shown that FABP4 deficiency ameliorates insulin resistance and prevents atherosclerosis in apolipoprotein E-deficient mice (10, 11). It remains unknown whether FABP4 is correlated with leptin or adiponectin in early life. Human studies have associated elevated circulating FABP4 levels with obesity, insulin resistance, type 2 diabetes (12, 13). It remains unclear whether GDM affects fetal FABP4 levels. We are aware of only three studies on the association between GDM and fetal/cord blood FABP4, and the findings have been inconsistent (7, 14, 15). The discrepant results may be partly related to the small to moderate sample sizes in these studies (GDM, all n<100), and thus the relative vulnerability to chance findings.

Females are more insulin resistant than males at birth (16). Clinical studies have reported higher circulating FABP4 levels in females *vs*. males in adulthood (17, 18). It is unknown whether any association between GDM and fetal circulating FABP4 may vary by sex. Sex hormones (estradiol and testosterone) play a critical role in fat deposition contributing to sex difference in insulin resistance (19, 20), and have been associated with the risks of metabolic syndrome and type 2 diabetes (21, 22). It is unknown whether sex hormones may be related to any potential sex dimorphic association between GDM and fetal FABP4.

In view of the above-discussed knowledge gaps, we sought to evaluate the association between GDM and cord blood FABP4, and explore potential sex dimorphic associations and the roles of sex hormones.

## Methods

### Study Design, Subjects, and Specimens

This was a nested matched case control study based on the recently developed Shanghai Birth Cohort (SBC) (23). In the SBC cohort, a total of 4127 pregnant women at preconception or early pregnancy care were recruited from six urban university affiliated tertiary obstetric care hospitals in Shanghai between 2013 and 2016. The women were followed up at the second and third trimesters of pregnancy and delivery. Data and specimens were collected at each study visit. All collected cord blood samples were kept on ice, stored temporarily in a 4°C refrigerator and centrifuged within 2 hours after the specimen collection. Serum and EDTA plasma samples were stored in multiple aliquots at −80°C until assays. The study was approved by the research ethics boards of Shanghai Xinhua Hospital (the coordination center, approved on August 23, 2013, ref no. M2013-010) and all participating hospitals. Written informed consent was obtained from all study participants.

GDM was diagnosed according to the International Association of Diabetes and Pregnancy Study Groups(IADPSG)criteria (24); if any one of the blood glucose values was at or above the following thresholds in the 75 g oral glucose tolerance test at 24-28 weeks of gestation: fasting 5.1 mmol/L, 1-hour 10.0 mmol/L and 2-hour 8.5 mmol/L.

As part of the SBC project, we conducted a nested case control study on the impacts of GDM on early life metabolic health biomarkers in the offspring (25, 26). Cases were the newborns of GDM mothers, and controls were the newborns of euglycemic mothers. Cases (n=153) and controls (n=153) were matched (1:1) by infant sex (the same) and gestational age at delivery (within 1 week) (25). Here, we reported the study data on cord blood FABP4 and sex hormones.

### Biochemical Assays

In all biomarker assays, the laboratory technicians were blinded to the clinical status (GDM or not) of study subjects. Cord plasma FABP4 was measured by an ELISA kit (R&D systems, Minnesota, USA). Plasma estradiol was measured by an ELISA kit (Labor Diagnostika Nord, Germany). Plasma testosterone was measured by a chemiluminescence immunoassay kit on a UniCel DXI 800 Access Immunoassay System (Beckman Coulter, USA). The detection limits were 6.55 pg/mL for FABP4, 6.2 pg/mL for estradiol, and 0.35 nmol/L for testosterone, respectively. The intra-assay and inter-assay coefficients of variation were in the ranges of 3.4-12.7% for FABP4, 3.1-6.4% for estradiol, and 1.7-7.1% for testosterone, respectively.

As reported previously, cord plasma leptin was measured by an ELISA kit from Invitrogen (Carlsbad, CA, USA), total and high-molecular-weight (HMW) adiponectin by an ELISA kit from ALPCO (Salem, NH, USA) (26). The intra-assay and inter-assay coefficients of variation were in the range of 6.9-10.4% (26).

### Statistical Analysis

Data are presented as Mean ± SD (standard deviation) and median (interquartile range) for continuous variables, and frequency (percentage) for categorical variables. Paired t-test was used in comparisons of continuous variables, and McNemar’s Chi-Square test was used in comparisons of dichotomous variables between GDM and matched controls. Log-transformed biomarker data were used in t tests, correlation and regression analyses. Pearson partial correlation coefficients were calculated to evaluate the correlations between biomarkers adjusting for gestational age at delivery/cord blood sampling. Fisher’s z test was used in comparisons of correlation coefficients between groups. Generalized linear models were applied to assess the differences in cord blood FABP4, estradiol and testosterone concentrations by GDM status or infant sex controlling for maternal and neonatal characteristics, and to assess the predictors of cord blood FABP4, and in tests for interactions. Maternal characteristics included age, ethnicity, education, parity, smoking or alcohol use during pregnancy, pre-pregnancy BMI (kg/m^2^), gestational hypertension, family history of diabetes, family history of hypertension. Neonatal characteristics included infant sex, gestational age, birth weight z score [according to the 2015 Chinese sex- and gestational age-specific birthweight standards (27)] and mode of delivery (cesarean section/vaginal). Matching variables were excluded (gestational age and infant sex) in the comparisons between GDM and control groups. Only co-variables with P<0.2 were included in the parsimonious final regression models to obtain more stable effect estimates. Mediation analyses were conducted to test whether sex hormones may mediate any relationship between GDM and cord plasma FABP4 using the product (“Baron and Kenney”) method (28). All statistical analyses were performed using SAS V.9.4 (SAS Institute, Cary, NC, USA). P value <0.05 was considered statistically significant in testing the difference in the primary outcome (cord plasma FABP4 concentration) between GDM and control groups. Using an online sample size and power calculator tool (http://powerandsamplesize.com/Calculators/), we calculated that with the study sample sizes (153 GDM-control pairs; 70 pairs of female newborns, 83 pairs of male newborns) and type 1 error (alpha) at 5%, the study had a power of 99% to detect a 0.5 SD or greater difference in a cord blood biomarker between GDM and control groups, and a power of >84% in sex-specific analyses.

## Results

### Maternal and Neonatal Characteristics

Maternal and neonatal characteristics of study subjects in this matched case control study in the Shanghai Birth Cohort have been described recently (25). Briefly, there were no significant differences in maternal age, education, parity, family history of diabetes, smoking or alcohol use in pregnancy. Maternal pre-pregnancy BMI was higher (mean: 23.6 *vs*. 21.6 kg/m^2^), gestational hypertension (5.2% *vs*. 0.6%) and cesarean section (57% *vs*. 36%) were more frequent in GDM *vs*. euglycemic pregnancies (all P<0.05). Birth weight z scores were higher in GDM *vs*. euglycemic pregnancies (P=0.04). Of the 306 newborns, 166 were males (83 GDM and 83 euglycemic mothers), and 140 were females (70 GDM and 70 euglycemic mothers). There were 142 cesarean section deliveries (97 elective and 45 emergency cesarean sections).

### Cord Plasma FABP4, Testosterone, and Estradiol Concentrations

Adjusting for maternal and neonatal characteristics, cord plasma FABP4 and testosterone concentrations were not significantly different between GDM and euglycemic pregnancies overall, while estradiol concentrations (49.0 ± 25.6 *vs*. 45.1 ± 23.6 ng/mL) were significantly higher in GDM pregnancies (**Table 1**). Descriptive statistics on cord plasma leptin, total and adiponectin concentrations have been reported recently (26).

**Table 1:** Cord plasma FABP4, estradiol and testosterone concentrations in the newborns of GDM *vs*. euglycemic (control) mothers.

Cord plasma	GDM (n=153)	Control (n=153)	Crude P*	Adjusted P*
FABP4	25.4 ± 18.3	22.0 ± 16.4	0.12	0.19
(ng/mL)	20.2 (12.3, 30.5)	16.7 (12.6, 24.1)		
Estradiol	49.0 ± 25.6	45.1 ± 23.6	0.10	**0.002**
(ng/mL)	41.2 (32.6, 59.4)	38.3 (30.6, 52.7)		
Testosterone	5.1 ± 1.1	5.0 ± 1.1	0.45	0.24
(nmol/L)	5.0 (4.5, 5.6)	4.9 (4.3, 5.5)		

Data presented are mean ± SD and median (inter-quartile range).

GDM, gestational diabetes mellitus; FABP-4, fatty acid binding protein 4;

*Crude P values were from paired t-tests in log-transformed biomarker data. Adjusted P values were from generalized linear models in the comparisons of log-transformed biomarker data adjusting for maternal (pre-pregnancy BMI, family history of diabetes, family history of hypertension, gestational hypertension) and neonatal (cesarean section) characteristics; other factors were excluded since they were similar and did not affect the comparisons between the two groups (all P>0.2). P values in bold, P<0.05.

In sex stratified analyses adjusting for maternal and neonatal characteristics, cord plasma FABP4 concentrations were higher in GDM *vs*. euglycemic pregnancies in males (27.0 ± 19.6 *vs*. 18.8 ± 9.61 ng/mL, P=0.045), but similar in females (23.2 ± 16.4 *vs*. 25.6 ± 21.2 ng/mL, P=0.60) (test for interaction, P=0.039) (**Table 2**). GDM was associated with higher cord plasma estradiol concentrations in males (P=0.005), but not in females (P=0.56) (test for interaction, P=0.052).

**Table 2:** Cord plasma FABP4, testosterone and estradiol concentrations in the newborns of GDM *vs*. euglycemic (control) mothers stratified by infant sex.

	Male newborns		Crude P*	Adjusted P*	Female newborns		Crude P*	Adjusted P*
	GDM	Control			GDM	Control		
**FABP4**	27.0 ± 19.6	18.8 ± 9.6	**0.007**	**0.045**	23.2 ± 16.4	25.6 ± 21.2	0.71	0.60
**(ng/mL)**								
**Testosterone**	5.2 ± 1.3	5.0 ± 0.9	0.45	0.37	4.9 ± 0.8	4.9 ± 1.2	0.81	0.20
**(nmol/L)**								
**Estradiol**	52.0 ± 28.6	44.2 ± 26.6	**0.02**	**0.005**	45.3 ± 20.8	46.0 ± 19.7	0.59	0.56
**(ng/mL)**								

Data presented are Mean ± SD. There were 166 male newborns (of 83 GDM and 83 euglycemic mothers) and 140 female newborns (of 70 GDM and 70 euglycemic mothers).

GDM, gestational diabetes mellitus; FABP4, fatty acid binding protein 4.

*Crude P values were from paired t-tests in log-transformed data. Adjusted P values were from generalized linear models in the comparisons of log-transformed biomarker data between the two groups adjusting for maternal (pre-pregnancy BMI, family history of diabetes, family history of hypertension, gestational hypertension) and neonatal (cesarean section) characteristics; other factors were excluded since they were similar and did not affect the comparisons (all P>0.2). P values in bold, P<0.05.

Tests for interaction between fetal sex and GDM: P=0.039 in the association with FABP4, P= 0.052 in the association with estradiol.

There were no significant differences in cord plasma testosterone concentrations between GDM and euglycemic pregnancies in both males and females. There were no sex differences in cord plasma FABP4, estradiol and testosterone concentrations in both GDM and euglycemic pregnancies (all P>0.05, **Table 3**).

**Table 3:** Cord plasma FABP4, testosterone and estradiol concentrations comparing male *vs*. female newborns in GDM and euglycemic (control) pregnancies.

	GDM		P*	Control		P*
	Male newborns	Female newborns		Male newborns	Female newborns	
**FABP4**	27.0 ± 19.6	23.2 ± 16.4	0.21	18.8 ± 9.6	25.6 ± 21.2	0.10
**(ng/mL)**						
**Testosterone**	5.2 ± 1.3	4.9 ± 0.8	0.41	5.0 ± 0.9	4.9 ± 1.2	0.40
**(nmol/L)**						
**Estradiol**	52.0 ± 28.6	45.3 ± 20.8	0.15	44.2 ± 26.6	46.0 ± 19.7	0.18
**(ng/mL)**						

GDM, gestational diabetes mellitus; FABP4, fatty acid binding protein 4.

Data presented are mean ± SD. There were 153 newborns (83 boys and 70 girls) of GDM and 153 newborns (83 boys and 70 girls) of euglycemic (control) mothers in the analyses.

*Crude P values in the comparisons of log-transformed biomarker data between male and female newborns; no adjustments were made since all co-variables (maternal and neonatal characteristics) were similar in male and female newborns (all P>0.2) and did not affect the comparisons.

### Correlations

**Figure 1:**
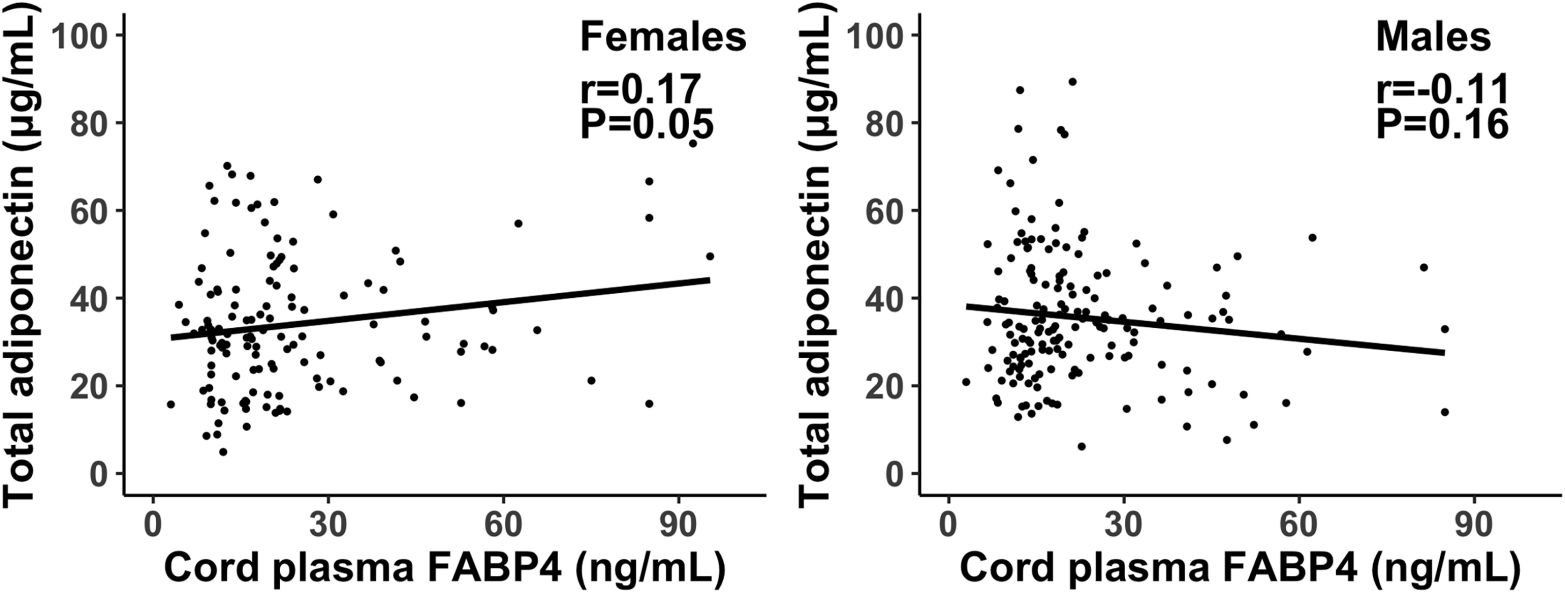
Scatter plots illustrating the different correlations of cord plasma FABP4 with total adiponectin in female and male newborns; the interpolation represents the regression line. P=0.02 in Fisher’s z test for difference in the correlation coefficients in males and females.

Cord plasma FABP4 was negatively correlated with gestational age at delivery (r=-0.16, P=0.01). Adjusting for gestational age at blood sampling, cord plasma FABP4 was positively correlated to adiponectin in females (r=0.17, P=0.053), but the correlation was in the opposite direction in males (r=-0.11, P=0.16) (Fisher’s z test for difference in correlation coefficients, P=0.02) (**Table 4**, **Figure 1**). Cord plasma FABP4 was positively correlated with birth weight z score, but was not correlated with estradiol, testosterone and leptin in both males and females. Cord plasma FABP4 was not correlated to leptin or adiponectin in GDM or euglycemic pregnancies (**Table 5**).

**Table 4:** Cord blood FABP4 in correlations with testosterone, estradiol, leptin, adiponectin and birth weight (z score) in males and females.

	All		Males		Females		*P for difference
	r	P	r	P	r	P	
Testosterone	-0.07	0.25	**-**0.07	0.39	-0.07	0.44	1.00
Estradiol	0.07	0.22	0.10	0.20	0.04	0.69	0.58
Leptin	0.09	0.15	0.045	0.58	0.14	0.12	0.44
Total adiponectin	0.03	0.61	-0.11	0.16	0.17	0.053	**0.02**
HMW adiponectin	0.02	0.76	-0.12	0.12	0.14	0.12	**0.03**
Birth weight z score	0.20	**<0.001**	0.22	**0.01**	0.19	**0.03**	0.80

FABP4, fatty acid binding protein 4; HMW, high molecular weight.

Data presented are Pearson partial correlation coefficients in log-transformed biomarker data adjusting for gestational age at delivery/cord blood sampling.

*P values in Fisher’s z tests for differences in correlation coefficients in males and females. P values in bold, P<0.05.

**Table 5:** Cord blood FABP4 in correlations with testosterone, estradiol, leptin, adiponectin and birth weight z score in GDM and euglycemic (control) pregnancies.

	GDM		Control		*P for difference
	r	P	r	P	
Testosterone	-0.14	0.11	0.02	0.81	0.19
Estradiol	0.02	0.82	0.12	0.16	0.41
Leptin	0.08	0.35	0.11	0.19	0.79
Total adiponectin	-0.06	0.46	0.16	0.052	0.06
HMW adiponectin	-0.05	0.52	0.15	0.07	0.08
Birth weight z score	0.22	0.01	0.15	0.06	0.55

GDM, gestational diabetes mellitus; FABP4, fatty acid binding protein 4; HMW, high molecular weight.

Data presented are Pearson partial correlation coefficients adjusting for gestational age at delivery/cord blood sampling. Log-transformed biomarker data were used in the analyses.

*P values in Fisher’s z tests for differences in the correlation coefficients in GDM and control groups.

### Determinants of Cord Plasma FABP4 Levels

There was a significant interaction between GDM and fetal sex in relation to cord plasma FABP4 (P=0.039). GDM was associated with a 22.5% (95% CI: 2.3-46.6%) increase in cord plasma FABP4 in males (P=0.03), but there was no association in females (**Table 6**). Higher cord plasma FABP4 levels were associated with family history of hypertension, cesarean section delivery and higher birth weight z scores. Cord plasma FABP4 concentrations were higher in either elective cesarean sections (27.7 ± 19.2 ng/mL, P<0.001) or emergency cesarean sections (31.1 ± 24.2 ng/mL, P<0.001) as compared to vaginal deliveries (18.8 ± 10.6 ng/mL). Higher gestational ages at delivery were associated with lower cord plasma FABP4 concentrations. Other factors were not associated with cord plasma FABP4, including family history of diabetes, maternal age, pre-pregnancy BMI, parity, education, smoking, alcohol use and fetal sex (all P>0.2). There were no significant interactions between GDM and pre-pregnancy BMI or maternal age in relation to cord plasma FABP4 (all P>0.05).

**Table 6:** Determinants of cord plasma FABP4 concentrations.

	All	P	Males	P	Females	P
	β (95% CI)		β (95% CI)		β (95% CI)	
GDM	9.4 (-4.8, 25.7)	0.21	22.5 (2.3, 46.6)	**0.03**	-5.4 (-24.0, 17.8)	0.62
FH of hypertension	17.5 (2.2, 35.2)	**0.02**	17.8 (-1.8, 41.3)	0.08	19.6 (-3.9, 48.8)	0.11
Gest. hypertension	-17.0 (-43.4, -21.7)	0.34	-2.6 (-36.0, 48.4)	0.90	-54.3 (-80.2, 5.5)	0.07
Cesarean section	28.8 (12.3, 47.7)	**<0.001**	22.3 (2.7, 45.6)	**0.02**	35.6 (8.6, 69.2)	**0.01**
Gestational age	-7.0 (-11.8, -1.9)	**0.01**	-6.8 (-12.8, -0.5)	**0.04**	-6.6 (-14.6, 2.1)	0.13
Birth weight z score	8.2 (1.7, 15.2)	**0.01**	9.2 (0.7, 18.5)	**0.03**	6.0 (-3.8, 16.8)	0.24

GDM, gestational diabetes mellitus; FABP4, fatty acid binding protein 4; FH, family history; Gest., gestational.

Data presented are the percentage change (95% CI) from generalized linear models, based on the regression coefficients for the outcome (FABP4 concentration) in log-transformed data. Only predictors with P<0.2 in predicting the outcome in at least one sex (male or female) group were retained in the final models. For consistency, the same set of predictors were retained in the final models for both sexes. Test for interaction with infant sex was significant for GDM only (P=0.04). Therefore, the primary effect estimates should be sex-specific for GDM, and be the effect estimates in the pooled total sample for other predictors.

P values in bold, P<0.05.

### Mediation Analyses

The positive association between GDM and cord plasma FABP4 in males was partly mediated by estradiol; the mediation effect was a 7.0% (95% CI: 0.6-13.7%, P=0.03) increase in cord plasma FABP4. In contrast, there was no mediation effect by testosterone (P=0.72). The positive relationship between cord plasma FABP4 and total adiponectin in females was not mediated by estradiol (P=0.35) or testosterone (P=0.98).

## Discussion

### Main Findings

The study is the first to demonstrate sex dimorphic associations between GDM and cord plasma FABP4 or estradiol, and between cord plasma FABP4 and adiponectin. GDM was associated with elevated cord plasma FABP4 and estradiol concentrations in males only, and FABP4 was positively correlated with adiponectin in females only. The positive association between GDM and cord plasma FABP4 in males appears to be partly mediated by estradiol.

### Data Interpretation and Comparisons to Findings in Previous Studies

Adipokines may be involved in the pathophysiology of GDM and its post-partum consequences (29). FABP4 has been associated with insulin resistance (13, 30). Studies have reported inconsistent findings on cord blood FABP4 levels in GDM (7, 14, 15). Two studies reported higher cord blood FABP4 levels (14, 15), while another study reported lower cord blood FABP4 levels in GDM *vs*. euglycemic pregnancies (7). Sample sizes in GDM pregnancies were 98, 50 and 26 in the three studies, respectively (7, 14, 15). In contrast, our study with a much larger sample size (153 GDM pregnancies) showed that GDM was associated with higher cord plasma FABP4 concentrations in males only. We could not reconcile our data against previous studies which did not report sex specific data. It should be noted that the difference would not be detected in the pooled total sample (males+females). Our comparisons were adjusted for maternal and neonatal characteristics, while two previous studies did not adjust for these characteristics (14, 15), and the other study adjusted for pre-pregnancy BMI and infant sex only (7). GDM has been associated with lower cord plasma adiponectin concentrations in females only (26). The current study adds new evidence suggesting a sex dimorphic impact of GDM on circulating levels of certain adipokines in early life.

Studies in adults have reported higher circulating FABP4 levels in women *vs*. men (17, 18). Androgen may contribute to such a sex difference through affecting body fat content as fat mass is positively correlated with circulating FABP4 levels (18). Circulating testosterone and FABP4 concentrations are negatively correlated in men, but positively correlated in women (18). However, we did not observe any association between cord plasma testosterone and FABP4 in newborns. Higher cord plasma FABP4 concentrations were observed in GDM *vs*. euglycemic pregnancies in males only, suggesting that GDM may up-regulate FABP4 expression/secretion in males during fetal life. Mediation analyses indicate that this male specific positive association between GDM and cord plasma FABP4 might be partly mediated by estradiol, but not related to testosterone. A study in muscle (myotube) cells suggested that estradiol could up-regulate FABP4 expression (31). This might explain the mediation effect of estradiol on higher FABP4 levels associated with GDM in male newborns. Further studies are warranted to validate whether this is a phenomenon unique to males, and to elucidate the underlying mechanisms.

Adipose tissue produces a number of adipokines that modulate insulin response (29). There is a lack of data on the relationship between FABP4 and other adipokines in early life. Our study is the first to reveal a sex dimorphic association between cord plasma FABP4 and adiponectin. Cord plasma FABP4 and adiponectin were positively correlated in females only, and the association was unrelated to sex hormones. This novel observation requires confirmation in more independent studies.

Elective cesarean delivery is a less stressful procedure to the fetus than vaginal delivery. Cord blood cortisol levels are lower in elective cesarean deliveries compared to vaginal deliveries (32). About 50% cesarean deliveries are elective cesarean sections in Shanghai (33). Such high rates of elective cesarean sections are common in China due to maternal preference and financial incentives for hospitals (34). A study in mice reported that dexamethasone (a synthetic glucocorticoid) injection increased FABP4 mRNA expression (35), and there have been no data in humans. We observed that cord plasma FABP4 levels were higher in cesarean deliveries (elective or not) *vs*. vaginal deliveries, even though fetal cortisol levels should be lower in elective cesarean deliveries. Further studies in other independent cohorts are required to confirm this new finding.

Elevated circulating FABP4 levels have been associated with a family history of hypertension (36). Consistent with this report, we observed higher cord plasma FABP4 concentrations in subjects with a family history of hypertension. Maternal and cord blood FABP4 concentrations were not correlated, suggesting fetal tissues might be the main source of FABP4 in cord blood (7). Consistent with our data, birth weight z score was positively correlated with cord blood FABP4 levels, while gestational age was negatively correlated with cord blood FABP4 levels in previous studies (37, 38). Fetal FABP4 may be expressed at higher levels in earlier gestational ages.

Elevated circulating estrogen levels have been related to insulin resistance in pregnancy (39). After binding to the estrogen receptor, estradiol may decrease insulin sensitivity through reducing the expression of the insulin sensitive membrane transporter - glucose transporter 4 in adipose tissue and muscle (40, 41). Two studies on cord blood estradiol levels in GDM showed inconsistent findings (42, 43). Qi et al. reported decreased cord blood estradiol levels in GDM pregnancies (n=204) (42), while Jin et al. observed no significant changes in GDM pregnancies (n=48) (43). In contrast, our data showed that GDM was associated with higher cord blood estradiol concentrations in males only. The reasons for these inconsistent findings are unclear, and may be partly due to the differences in sample size and GDM diagnostic criteria. More studies in larger cohorts are warranted to clarify the association.

### Strengths and Limitations

The study was based on a large birth cohort. Biochemical assays were of high quality, and study subject’s clinical status was blinded to assay technicians. The study has some limitations. We could not draw conclusions regarding causality due to the observational nature of the study. The study subjects were all Chinese. More studies in other populations are required to determine the generalizability of the study findings to other ethnic groups.

## Conclusion

Our study data suggest a sex dimorphic impact of GDM on FABP4 and estradiol levels in early life in the offspring. The male specific positive association between GDM and FABP4 appears to be partly mediated by estradiol. There may be a sex dimorphic association between FABP4 and adiponectin in early life. These new findings suggest the need for more research to illuminate the sex specific metabolic “programming” impact targets and long-term consequences that could guide the development of targeted programming interventions to reduce the vulnerability to insulin resistance and type 2 diabetes.

## Data Availability Statement

The datasets presented in this article are not readily available because access to the deidentified participant research data must be approved by the research ethics board on a case-by-case basis. Requests to access the datasets should be directed to the corresponding author (zcluo@lunenfeld.ca; feili@shsmu.edu.cn).

## Ethics Statement

The studies involving human participants were reviewed and approved by the research ethics committees of the coordination center (Xinhua Hospital, reference number M2013-010) and all participating hospitals. The patients/participants provided their written informed consent to participate in this study.

## Author Contributions

Z-CL, G-HZ, FL, JZ, and FO conceived the study. XL, TZ, Y-JX, M-NY, W-JW, RH, G-HZ, Y-NG, JZ, FO, FL, and Z-CL contributed to the acquisition of research data. XL and TZ conducted the literature review, data analysis, and drafted the manuscript. All authors contributed to the article and approved the submitted version.

## Funding

This work was supported by research grants from the Ministry of Science and Technology of China (2019YFA0802501, 2017YFE0124700), the Shanghai Municipal Health Commission (2020CXJQ01), the Shanghai Science and Technology Commission (19410713500, 21410713500), the National Natural Science Foundation of China (81961128023, 81903323, 81761128035 and 81930095), the National Human Genetic Resources Sharing Service Platform (2005DKA21300), and the Canadian Institutes of Health Research (158616). The funders have no role in all aspects of the study, including study design, data collection and analysis, the preparation of the manuscript and the decision for publication.

## Conflict of Interest

The authors declare that the research was conducted in the absence of any commercial or financial relationships that could be construed as a potential conflict of interest.

The reviewer HH declared a shared affiliation, with no collaboration, with one of the authors, XL, to the handling editor at the time of review.

## Publisher’s Note

All claims expressed in this article are solely those of the authors and do not necessarily represent those of their affiliated organizations, or those of the publisher, the editors and the reviewers. Any product that may be evaluated in this article, or claim that may be made by its manufacturer, is not guaranteed or endorsed by the publisher.

## Abbreviations

FABP4, Fatty acid binding protein 4; GDM, Gestational diabetes mellitus; HMW, high-molecular-weight; IADPSG, International Association of Diabetes and Pregnancy Study Groups; SBC, Shanghai Birth Cohort.

## References

[1] American Diabetes A. Erratum. Classification and Diagnosis of Diabetes. Sec. 2. In Standards of Medical Care in Diabetes-2016. Diabetes Care (2016) 39(Suppl. 1):S13–22. doi: 10.2337/dc16-er09 27555625

[2] Melchior H Kurch-Bek D Mund M. The Prevalence of Gestational Diabetes. Dtsch Arztebl Int (2017) 114(24):412–8. doi: 10.3238/arztebl.2017.0412 PMC5499505 28669379

[3] Egeland GM Meltzer SJ. Following in Mother's Footsteps? Mother-Daughter Risks for Insulin Resistance and Cardiovascular Disease 15 Years After Gestational Diabetes. Diabetes Med (2010) 27(3):257–65. doi: 10.1111/j.1464-5491.2010.02944.x 20536487

[4] Metzger BE. Long-Term Outcomes in Mothers Diagnosed With Gestational Diabetes Mellitus and Their Offspring. Clin Obstet Gynecol (2007) 50(4):972–9. doi: 10.1097/GRF.0b013e31815a61d6 17982340

[5] Bao W Baecker A Song Y Kiely M Liu S Zhang C. Adipokine Levels During the First or Early Second Trimester of Pregnancy and Subsequent Risk of Gestational Diabetes Mellitus: A Systematic Review. Metabolism (2015) 64(6):756–64. doi: 10.1016/j.metabol.2015.01.013 PMC4625979 25749468

[6] Okereke NC Uvena-Celebrezze J Hutson-Presley L Amini SB Catalano PM. The Effect of Gender and Gestational Diabetes Mellitus on Cord Leptin Concentration. Am J Obstet Gynecol (2002) 187(3):798–803. doi: 10.1067/mob.2002.125887 12237665

[7] Ortega-Senovilla H Schaefer-Graf U Meitzner K Abou-Dakn M Graf K Kintscher U. Gestational Diabetes Mellitus Causes Changes in the Concentrations of Adipocyte Fatty Acid-Binding Protein and Other Adipocytokines in Cord Blood. Diabetes Care (2011) 34(9):2061–6. doi: 10.2337/dc11-0715 PMC3161255 21775757

[8] Trojnar M Patro-Małysza J Kimber-Trojnar Ż Leszczyńska-Gorzelak B Mosiewicz J. Associations Between Fatty Acid-Binding Protein 4⁻A Proinflammatory Adipokine and Insulin Resistance, Gestational and Type 2 Diabetes Mellitus. Cells (2019) 8(3):227. doi: 10.3390/cells8030227 PMC6468522 30857223

[9] Luo ZC Delvin E Fraser WD Audibert F Deal CI Julien P. Maternal Glucose Tolerance in Pregnancy Affects Fetal Insulin Sensitivity. Diabetes Care (2010) 33(9):2055–61. doi: 10.2337/dc10-0819 PMC2928362 20573751

[10] Makowski L Boord JB Maeda K Babaev VR Uysal KT Morgan MA. Lack of Macrophage Fatty-Acid-Binding Protein Ap2 Protects Mice Deficient in Apolipoprotein E Against Atherosclerosis. Nat Med (2001) 7(6):699–705. doi: 10.1038/89076 11385507 PMC4027052

[11] Boord JB Maeda K Makowski L Babaev VR Fazio S Linton MF. Combined Adipocyte-Macrophage Fatty Acid-Binding Protein Deficiency Improves Metabolism, Atherosclerosis, and Survival in Apolipoprotein E-Deficient Mice. Circulation (2004) 110(11):1492–8. doi: 10.1161/01.Cir.0000141735.13202.B6 PMC4027050 15353487

[12] Tso AW Xu A Sham PC Wat NM Wang Y Fong CH. Serum Adipocyte Fatty Acid Binding Protein as a New Biomarker Predicting the Development of Type 2 Diabetes: A 10-Year Prospective Study in a Chinese Cohort. Diabetes Care (2007) 30(10):2667–72. doi: 10.2337/dc07-0413 17620449

[13] Xu A Wang Y Xu JY Stejskal D Tam S Zhang J. Adipocyte Fatty Acid-Binding Protein is a Plasma Biomarker Closely Associated With Obesity and Metabolic Syndrome. Clin Chem (2006) 52(3):405–13. doi: 10.1373/clinchem.2005.062463 16423904

[14] Patro-Małysza J Trojnar M Kimber-Trojnar Ż Mierzyński R Bartosiewicz J Oleszczuk J. FABP4 in Gestational Diabetes-Association Between Mothers and Offspring. J Clin Med (2019) 8(3):285. doi: 10.3390/jcm8030285 PMC6462903 30818771

[15] Zhang Y Lu JH Zheng SY Yan JH Chen L Liu X. Serum Levels of Nesfatin-1 are Increased in Gestational Diabetes Mellitus. Gynecol Endocrinol (2017) 33(8):621–4. doi: 10.1080/09513590.2017.1306849 28361552

[16] Shields BM Knight B Hopper H Hill A Powell RJ Hattersley AT. Measurement of Cord Insulin and Insulin-Related Peptides Suggests That Girls are More Insulin Resistant Than Boys at Birth. Diabetes Care (2007) 30(10):2661–6. doi: 10.2337/dc06-1501 17475939

[17] Djoussé L Khawaja O Bartz TM Biggs ML Ix JH Zieman SJ. Plasma Fatty Acid-Binding Protein 4, Nonesterified Fatty Acids, and Incident Diabetes in Older Adults. Diabetes Care (2012) 35(8):1701–7. doi: 10.2337/dc11-1690 PMC3402261 22584136

[18] Hu X Ma X Pan X Luo Y Xu Y Xiong Q. Association of Androgen With Gender Difference in Serum Adipocyte Fatty Acid Binding Protein Levels. Sci Rep (2016) 6:27762. doi: 10.1038/srep27762 27270834 PMC4897720

[19] Mongraw-Chaffin ML Anderson CA Allison MA Ouyang P Szklo M Vaidya D. Association Between Sex Hormones and Adiposity: Qualitative Differences in Women and Men in the Multi-Ethnic Study of Atherosclerosis. J Clin Endocrinol Metab (2015) 100(4):E596–600. doi: 10.1210/jc.2014-2934 PMC4399289 25636047

[20] Gates MA Mekary RA Chiu GR Ding EL Wittert GA Araujo AB. Sex Steroid Hormone Levels and Body Composition in Men. J Clin Endocrinol Metab (2013) 98(6):2442–50. doi: 10.1210/jc.2012-2582 PMC3667256 23626004

[21] Agirbasli M Agaoglu NB Orak N Caglioz H Ocek T Poci N. Sex Hormones and Metabolic Syndrome in Children and Adolescents. Metabolism (2009) 58(9):1256–62. doi: 10.1016/j.metabol.2009.03.024 19497594

[22] Rao PM Kelly DM Jones TH. Testosterone and Insulin Resistance in the Metabolic Syndrome and T2DM in Men. Nat Rev Endocrinol (2013) 9(8):479–93. doi: 10.1038/nrendo.2013.122 23797822

[23] Zhang J Tian Y Wang W Ouyang F Xu J Yu X. Cohort Profile: The Shanghai Birth Cohort. Int J Epidemiol (2019) 48(1):21–g. doi: 10.1093/ije/dyy277 30629180

[24] Metzger BE Gabbe SG Persson B Buchanan TA Catalano PA Damm P. International Association of Diabetes and Pregnancy Study Groups Recommendations on the Diagnosis and Classification of Hyperglycemia in Pregnancy. Diabetes Care (2010) 33(3):676–82. doi: 10.2337/dc09-1848 PMC2827530 20190296

[25] Wang WJ Zhang L Zheng T Zhang GH Du K Yang MN. Fetuin-A and Fetal Growth in Gestational Diabetes Mellitus. BMJ Open Diabetes Res Care (2020) 8(1):e000864. doi: 10.1136/bmjdrc-2019-000864 PMC7039609 32049636

[26] Yang MN Chiu HC Wang WJ Fang F Zhang GH Zhu H. Sex Dimorphism in the Associations of Gestational Diabetes With Cord Blood Adiponectin and Retinol-Binding Protein 4. BMJ Open Diabetes Res Care (2020) 8(1):e001310. doi: 10.1136/bmjdrc-2020-001310 PMC7517565 32973071

[27] Zhu L Zhang R Zhang S Shi W Yan W Wang X. Chinese Neonatal Birth Weight Curve for Different Gestational Age. Zhonghua Er Ke Za Zhi (2015) 53(2):97–103. doi: 10.3760/cma.j.issn.0578-1310.2015.02.007 25876683

[28] VanderWeele TJ. Mediation Analysis: A Practitioner's Guide. Annu Rev Public Health (2016) 37:17–32. doi: 10.1146/annurev-publhealth-032315-021402 26653405

[29] Fasshauer M Blüher M Stumvoll M. Adipokines in Gestational Diabetes. Lancet Diabetes Endocrinol (2014) 2(6):488–99. doi: 10.1016/s2213-8587(13)70176-1 24731659

[30] Tu WJ Guo M Shi XD Cai Y Liu Q Fu CW. First-Trimester Serum Fatty Acid-Binding Protein 4 and Subsequent Gestational Diabetes Mellitus. Obstet Gynecol (2017) 130(5):1011–6. doi: 10.1097/AOG.0000000000002310 29016489

[31] Berio E Divari S Starvaggi Cucuzza L Biolatti B Cannizzo FT. 17β-Estradiol Upregulates Oxytocin and the Oxytocin Receptor in C2C12 Myotubes. PeerJ (2017) 5:e3124. doi: 10.7717/peerj.3124 28382233 PMC5376115

[32] Słabuszewska-Jóżwiak A Włodarczyk M Kilian K Rogulski Z Ciebiera M Szymańska-Majchrzak J. Does the Caesarean Section Impact on 11β HSD2 and Fetal Cortisol? Int J Environ Res Public Health (2020) 17(15):5566. doi: 10.3390/ijerph17155566 PMC7432821 32752242

[33] Ji H Jiang H Yang L Qian X Tang S. Factors Contributing to the Rapid Rise of Caesarean Section: A Prospective Study of Primiparous Chinese Women in Shanghai. BMJ Open (2015) 5(11):e008994. doi: 10.1136/bmjopen-2015-008994 PMC4654304 26567254

[34] Mi J Liu F. Rate of Caesarean Section is Alarming in China. Lancet (2014) 383(9927):1463–4. doi: 10.1016/s0140-6736(14)60716-9 24766963

[35] Bose SK Hutson I Harris CA. Hepatic Glucocorticoid Receptor Plays a Greater Role Than Adipose GR in Metabolic Syndrome Despite Renal Compensation. Endocrinology (2016) 157(12):4943–60. doi: 10.1210/en.2016-1615 PMC5133352 27754788

[36] Ota H Furuhashi M Ishimura S Koyama M Okazaki Y Mita T. Elevation of Fatty Acid-Binding Protein 4 is Predisposed by Family History of Hypertension and Contributes to Blood Pressure Elevation. Am J Hypertens (2012) 25(10):1124–30. doi: 10.1038/ajh.2012.88 PMC3449332 22717543

[37] Joung KE Cataltepe SU Michael Z Christou H Mantzoros CS. Cord Blood Adipocyte Fatty Acid-Binding Protein Levels Correlate With Gestational Age and Birth Weight in Neonates. J Clin Endocrinol Metab (2017) 102(5):1606–13. doi: 10.1210/jc.2016-3831 PMC5443332 28324040

[38] Papathanasiou AE Briana DD Gavrili S Georgantzi S Papathoma E Marmarinos A. Cord Blood Fatty Acid-Binding Protein-4 Levels are Upregulated at Both Ends of the Birthweight Spectrum. Acta Paediatr (2019) 108(11):2083–8. doi: 10.1111/apa.14826 31025416

[39] Barros RP Morani A Moriscot A Machado UF. Insulin Resistance of Pregnancy Involves Estrogen-Induced Repression of Muscle GLUT4. Mol Cell Endocrinol (2008) 295(1-2):24–31. doi: 10.1016/j.mce.2008.07.008 18692545

[40] Ropero AB Alonso-Magdalena P Quesada I Nadal A. The Role of Estrogen Receptors in the Control of Energy and Glucose Homeostasis. Steroids (2008) 73(9-10):874–9. doi: 10.1016/j.steroids.2007.12.018 18249429

[41] Jambaldorj B Terada E Hosaka T Kishuku Y Tomioka Y Iwashima K. Cysteine String Protein 1 (CSP1) Modulates Insulin Sensitivity by Attenuating Glucose Transporter 4 (GLUT4) Vesicle Docking With the Plasma Membrane. J Med Invest (2013) 60(3-4):197–204. doi: 10.2152/jmi.60.197 24190036

[42] Qi X Gong B Yu J Shen L Jin W Wu Z. Decreased Cord Blood Estradiol Levels in Related to Mothers With Gestational Diabetes. Medicine (Baltimore) (2017) 96(21):e6962. doi: 10.1097/md.0000000000006962 28538390 PMC5457870

[43] Jin Z Guan X Gao H Shang L Gao M Su D. The Change in Sex Hormone Binding Globulin and the Influence by Gestational Diabetes Mellitus in Fetal Period. Gynecol Endocrinol (2009) 25(10):647–52. doi: 10.1080/09513590903015437 19557594

